# Gut microbiota dysbiosis in rheumatoid arthritis: mechanisms linking intestinal barrier dysfunction to synovial inflammation

**DOI:** 10.3389/fimmu.2026.1884535

**Published:** 2026-08-28

**Authors:** Shirin Hafezi, Fatemeh Saheb Sharif-Askari, Narjes Saheb Sharif-Askari, Mariam Wed Eladham, Ahmed S. Zayat, Rabih Halwani

**Affiliations:** 1 Research Institute for Medical and Health Sciences, University of Sharjah, Sharjah, United Arab Emirates; 2 Department of Pharmacy Practice and Pharmacotherapeutics, College of Pharmacy, University of Sharjah, Sharjah, United Arab Emirates; 3 Department of Clinical Sciences, College of Medicine, University of Sharjah, Sharjah, United Arab Emirates; 4 Rheumatology Department, University Hospital Sharjah, University of Sharjah, Sharjah, United Arab Emirates; 5 Health and Wellbeing Sector, NEOM, Tabuk, Saudi Arabia; 6 King Saud University Celiac Disease Research Chair, Department of Pediatrics, College of Medicine, King Saud University, Riyadh, Saudi Arabia; 7 Prince Fahad Bin Sultan Chair for Biomedical Research, University of Tabuk, Tabuk, Saudi Arabia; 8 College of Medicine, Alfaisal University, Riyadh, Saudi Arabia

**Keywords:** gut microbiota, gut-joint axis, intestinal barrier dysfunction, rheumatoid arthritis, short-chain fatty acids, Th17/Treg imbalance

## Abstract

Rheumatoid arthritis (RA) is a chronic, systemic, multifactorial autoimmune disease characterized by persistent synovial inflammation and progressive joint destruction. Although its etiology is complex, accumulating evidence suggests that alterations in the gut microbiota may influence disease susceptibility and progression through interactions with the host immune system. Throughout this review, we discuss the gut microbiota as one component of a broader network of mechanisms contributing to RA pathogenesis rather than as the sole initiating factor. Compromised intestinal barrier integrity and microbial dysbiosis may facilitate the translocation of microbial products, promoting immune activation and loss of tolerance. These alterations have been proposed to contribute to several pathogenic mechanisms, including molecular mimicry, protein citrullination, and disruption of the Th17/Treg balance, thereby linking mucosal immune responses with synovial inflammation. Patients with RA also frequently exhibit reduced intestinal production of short-chain fatty acids (SCFAs), particularly butyrate, which is associated with decreased abundances of SCFA-producing bacteria, including *Faecalibacterium* and *Roseburia*, and lower fecal and circulating butyrate levels. Reduced SCFA availability may further exacerbate inflammatory pathways and impair immune homeostasis. In parallel, gut-imprinted immune cells may migrate to the joints through shared homing pathways, potentially amplifying local inflammation. Taken together, current evidence supports the gut–joint axis as a promising framework for understanding immune dysregulation in RA and highlights microbiota-targeted interventions as potential adjunctive therapeutic strategies.

## 1 Introduction

Rheumatoid arthritis (RA) is a chronic, systemic immune-mediated inflammatory disorder characterized by dysregulation of multiple signaling networks and immune cell populations, leading to progressive joint destruction, persistent synovial inflammation, and systemic complications (1, 2). Autoimmune diseases such as RA are associated with a combination of genetic, environmental, and immunological risk factors and may develop at any age; however, females are two to three times more susceptible than males (3).

Dysregulation of both innate and adaptive immunity, as well as stromal cells such as synovial fibroblasts, has been observed in RA (4). Increased circulating abundance of CD45RO^+^ memory CD4^+^ and CD8^+^ T cells expressing markers associated with immune exhaustion (PD-1, CTLA-4) and activation markers (CD38, Ki-67, ICOS) has been detected in RA and has been associated with disease activity and arthritis flares (4). Despite significant advances in understanding RA pathogenesis, many aspects of disease initiation and progression remain incompletely defined.

Increasing evidence suggests that immune dysregulation in RA may originate outside the joints, particularly at mucosal surfaces (5). Among the mucosal sites investigated, the gastrointestinal (GI) tract has received considerable attention. Dysbiosis of the gut microbiota has been implicated in the development of several autoimmune diseases, including RA (5). One proposed pathogenic model posits that interactions between a genetically predisposed immune system and an altered intestinal microbiota may contribute to the initiation and perpetuation of autoimmune responses. These early immune perturbations may precede clinical disease and subsequently transition to synovial involvement, thereby potentially linking mucosal immune activation with joint-specific inflammation (1). Accordingly, investigating the gut-joint axis provides important insights into the early immunological events that shape RA progression.

Despite growing evidence linking gut microbial dysbiosis to RA, the available evidence is predominantly based on cross-sectional human studies, limiting the ability to determine whether these microbial changes contribute to disease pathogenesis or arise as a consequence of chronic inflammation, treatment, or other disease-related factors. Experimental animal models, such as collagen-induced arthritis (CIA) and antigen-induced arthritis (AIA), have provided important mechanistic insights supporting a gut-to-joint pathway; however, they may not fully reflect human disease and therefore do not establish a unidirectional causal relationship between gut dysbiosis and joint inflammation. Recent reviews further emphasize that the relationship is likely bidirectional, with systemic inflammation and RA itself capable of reshaping gut microbial communities (6). Furthermore, host and environmental factors are well-recognized confounders in human microbiome studies and should be carefully considered when interpreting disease-associated microbial signatures. Factors such as body mass index (BMI) (7), diet (8), smoking (9), age (10), and geographic location (11) can independently influence gut microbiome composition, potentially confounding associations between microbial alterations and RA.

Medication use, particularly methotrexate, is also known to alter gut microbial composition. Emerging pharmacomicrobiome studies suggest that gut microbial communities can both influence and be altered by disease-modifying antirheumatic drug (DMARD) treatment, further complicating the interpretation of microbiome signatures in RA. Therefore, medication exposure and other potential confounding factors should be carefully considered when interpreting cross-sectional microbiome studies (7, 12). In a recent systematic review, Ng and Lassere (2025) investigated the gastrointestinal microbiome in RA, ankylosing spondylitis, psoriatic arthritis, and reactive arthritis. Although the authors found consistent evidence of gut microbial dysbiosis, including altered microbial diversity and composition compared with healthy controls, the predominantly cross-sectional and highly heterogeneous nature of the available data limited causal inference, making it difficult to determine whether microbiome alterations represent a cause or a consequence of disease and its treatment (13).

The GI tract harbors the majority of the body’s immune cells, which continuously interact with the gut microbiota to maintain immune tolerance toward beneficial bacteria (14, 15). Beneficial gut bacteria metabolize dietary fibers to produce short-chain fatty acids (SCFAs), particularly butyrate, which functions as a ligand for the G-protein-coupled receptor GPR109A expressed on intestinal immune cells. Upon binding to GPR109A, butyrate promotes IL-10 and retinoic acid production, thereby upregulating FOXP3 expression and driving differentiation of naïve T cells into regulatory t cells (Tregs), thereby supporting immune homeostasis (15).

Conversely, gut microbiota dysbiosis may compromise intestinal barrier integrity and disrupt immune regulation (16). Patients with RA have been shown to exhibit impaired intestinal barrier integrity, with decreased expression of the tight junction protein zonula occludens-1 (ZO-1) in the colon (17). Altered expression or loss of tight junction proteins increases intestinal permeability, a phenomenon commonly referred to as “leaky gut”, which has been reported in patients with RA (17).

Disruption of the intestinal barrier, together with microbial dysbiosis, may permit the translocation of microorganisms and microbial products into the lamina propria and subepithelial compartments, potentially leading to aberrant activation of innate immune cells and subsequent activation of autoreactive B and T cells (5, 18, 19). These events may promote systemic autoimmunity and contribute to joint inflammation characteristic of RA (5, 18).

Although accumulating evidence suggests an important role for the gut–joint axis in RA pathogenesis, the directionality of this relationship remains an area of active investigation. Rather than representing a strictly unidirectional gut-to-joint process, RA is increasingly recognized as a multifactorial disease in which systemic immune dysregulation, host genetics, environmental exposures, and mucosal inflammation interact in a dynamic and potentially bidirectional manner. Several recent reviews have comprehensively summarized the contributions of gut dysbiosis to RA, focusing on microbial compositional changes, immune dysregulation, and microbiota-derived metabolites (1, 2, 20, 21). However, a barrier-centered perspective describing how disruption of intestinal defense mechanisms may contribute to RA pathogenesis has received comparatively less attention. In this review, we propose a four-layer intestinal barrier framework as a conceptual model describing how disruption of intestinal barrier defenses may link gut dysbiosis with immune activation and the pathogenesis of RA. By considering these complementary barrier systems as an interconnected functional unit, we provide a structured mechanistic model illustrating the proposed relationships among microbial dysbiosis, intestinal barrier dysfunction, immune activation, systemic autoimmunity, and joint inflammation.

## 2 The gut barrier: a four-layer model of integrity

### 2.1 The luminal microbial and biochemical defense layer

The first layer of the intestinal barrier comprises the luminal microbial and biochemical defense system, which serves as the earliest interface between the host and the intestinal environment. This layer consists of the commensal gut microbiota together with soluble defense molecules, including intestinal alkaline phosphatase (IAP), antimicrobial peptides (AMPs), secretory immunoglobulin A (sIgA), bile acids, and other antimicrobial factors that collectively maintain microbial homeostasis, detoxify microbial products, and prevent excessive immune activation.

**Figure 1:**
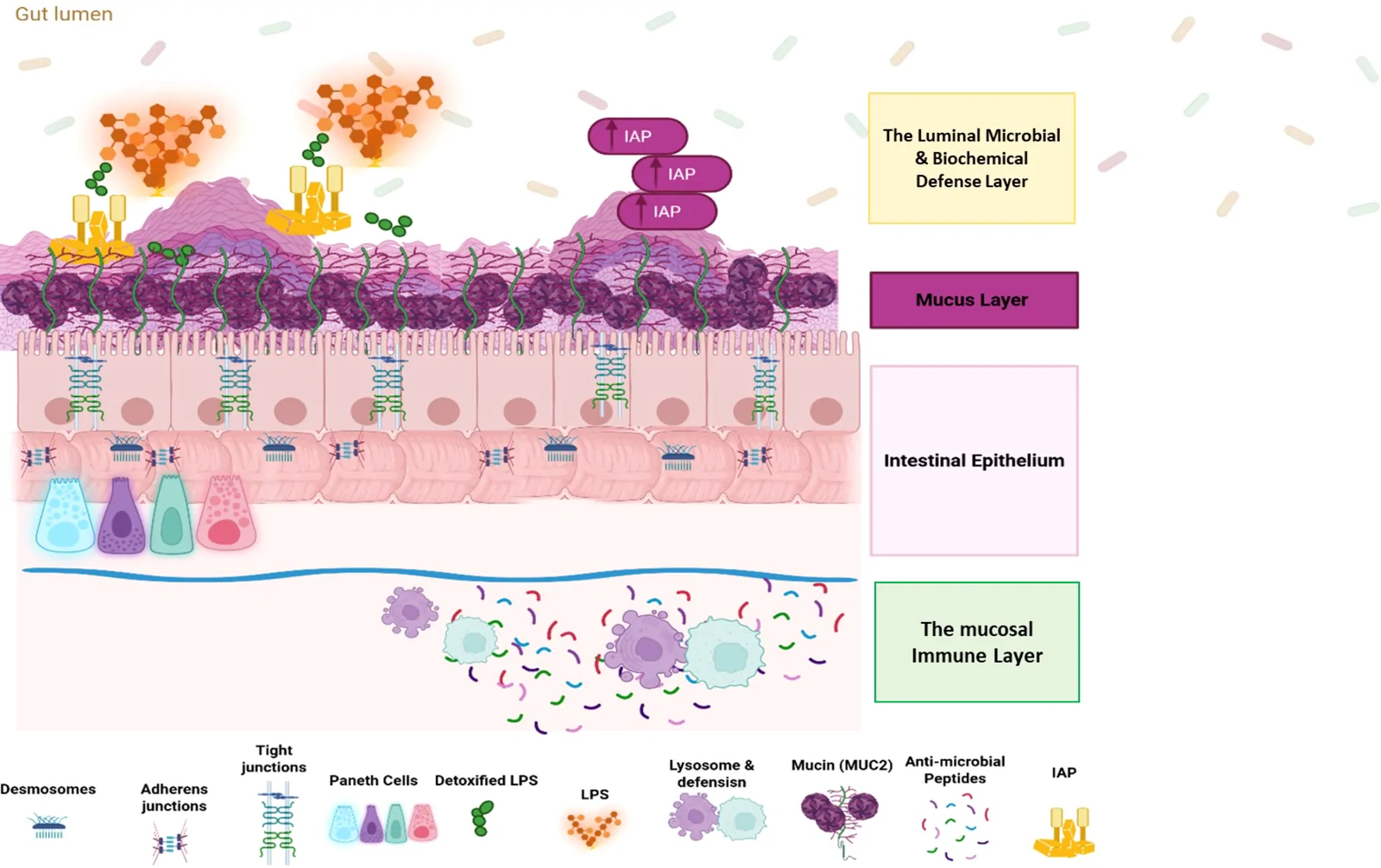
The four-layer model of intestinal barrier integrity. The gastrointestinal (GI) tract is continuously exposed to a dense and diverse microbial community derived from diet and the external environment. To maintain homeostasis while permitting nutrient absorption and immune tolerance, the intestine relies on a highly organized, multilayered barrier system that limits microbial encroachment and systemic dissemination of microbial products. This barrier comprises four functionally interconnected layers: (1) the luminal microbial and biochemical defense layer, consisting of the commensal gut microbiota together with soluble defense molecules, including intestinal alkaline phosphatase (IAP), antimicrobial peptides (AMPs), secretory immunoglobulin A (sIgA), bile acids, and other antimicrobial factors that collectively maintain microbial homeostasis and limit excessive immune activation; (2) the mucus layer, enriched in mucin (MUC2), which forms a physical barrier that spatially separates luminal microorganisms from the epithelial surface; (3) the intestinal epithelial layer, formed by tightly connected epithelial cells that regulate selective permeability through tight junctions, adherens junctions, and desmosomes; and (4) the mucosal immune layer, located primarily within the lamina propria and composed of innate and adaptive immune cells, including dendritic cells, macrophages, innate lymphoid cells, plasma cells, B cells, T cells, and regulatory T cells (Tregs), which maintain immune tolerance toward commensal microorganisms while mounting protective responses against pathogens. Coordinated interactions among these four layers preserve intestinal barrier integrity and immune homeostasis, whereas disruption of any layer promotes increased intestinal permeability, microbial translocation, immune dysregulation, and systemic inflammation. Created with BioRender.com.

Among these defense mechanisms, IAP, an enzyme secreted by intestinal epithelial cells into the gut lumen, plays an essential role in regulating intestinal lipid absorption, thereby supporting nutrient uptake and metabolic homeostasis. Beyond its metabolic functions, IAP is a critical component of innate host defense. It detoxifies bacterial endotoxins, such as lipopolysaccharide (LPS), through dephosphorylation, thereby reducing LPS-mediated activation of Toll-like receptor 4 (TLR4) and dampening downstream inflammatory signaling. In addition to LPS, IAP dephosphorylates other pathogen-associated molecular patterns (PAMPs), including flagellin and CpG DNA, further limiting excessive immune activation (22, 23). Reduced IAP activity has been associated with increased intestinal inflammation, microbial dysbiosis, and susceptibility to inflammatory and metabolic diseases (**Figure 1**).

### 2.2 The mucus layer: a physical and microbial interface

The second layer of the intestinal barrier is the mucus layer, which maintains homeostasis between the host and microbiota by segregating microorganisms from the epithelial surface (24). This layer is organized into two structurally distinct sublayers: an inner mucus layer tightly bound to the epithelial surface, which restricts bacterial contact with intestinal epithelial cells, and an outer mucus layer that secretes mucus (25, 26). Disruption of the mucus layer enables pathogen colonization of atypical intestinal niche (24). The principal structural component of this layer is mucin 2 (MUC2), secreted by goblet cells. Upon secretion, MUC2 forms a hydrated gel-like matrix that, together with other proteins, creates a well-organized mucus barrier. Disruption of this layer through excessive degradation or impaired mucin production compromises barrier integrity and predisposes the intestine to inflammation and disease (27) (**Figure 1**).

### 2.3 The intestinal epithelium: a selective cellular barrier

The third layer consists of the intestinal epithelial cell monolayer, which functions as a highly selective barrier that prevents the entry of foreign antigens and toxins while permitting the absorption of water, electrolytes, and dietary nutrients from the intestinal lumen into the systemic circulation. This selective permeability is maintained by specialized intercellular junctional complexes, including tight junctions, adherens junctions, and desmosomes. Tight junction proteins, such as zonula occludens (ZO) proteins and occludins, together with adherens junction proteins including cadherins and catenins, are critical for maintaining epithelial integrity and regulating paracellular transport (27–30). Disruption of these junctional complexes increases intestinal permeability (“leaky gut”), facilitating microbial translocation, immune activation, and inflammation (**Figure 1**).

### 2.4 The mucosal immune layer: immune surveillance and tolerance

The fourth layer of the intestinal barrier comprises the mucosal immune system, located primarily within the lamina propria, which provides continuous immune surveillance while maintaining tolerance toward commensal microorganisms and dietary antigens (14, 27). This layer contains innate and adaptive immune cells, including dendritic cells, macrophages, innate lymphoid cells, plasma cells, B cells, T cells, and Tregs, which collectively coordinate antimicrobial defense, immune regulation, and tissue homeostasis (14, 15, 27). Plasma cell-derived secretory immunoglobulin A (sIgA) contributes to mucosal defense by neutralizing microorganisms and preventing their adherence to the intestinal epithelium, while continuous crosstalk between immune cells and the gut microbiota promotes immune tolerance and limits excessive inflammation (14, 15). Disruption of mucosal immune homeostasis impairs immune tolerance, promotes chronic inflammation, and contributes to the development of immune-mediated diseases, including RA (15) (**Figure 1**).

## 3 Gut dysbiosis and the gut–joint axis in rheumatoid arthritis

Collectively, disruption of the four complementary layers of the intestinal barrier compromises mucosal homeostasis and facilitates the translocation of microbial products, including LPS, flagellin, and other pathogen-associated molecular patterns (PAMPs), beyond the intestinal lumen. These alterations may extend beyond localized intestinal inflammation by reshaping systemic immune signaling, altering T-cell polarization, and modifying the circulating metabolite milieu. Consequently, intestinal barrier dysfunction has been proposed as an important physiological link between gut-derived immune perturbations and extra-intestinal manifestations, including RA (31).

Specifically, reduced IAP activity may impair endotoxin detoxification, while mucus thinning and reduced mucin production diminish the physical separation between luminal microorganisms and the epithelial surface. In parallel, disruption of tight and adherens junctions increases paracellular permeability (“leaky gut”), allowing greater access of microbial products to the underlying mucosa. Impairment of Paneth cell- derived AMPs further promotes the expansion of pathobionts and microbial imbalance. Collectively, these alterations are thought to influence systemic immune homeostasis and may contribute to joint inflammation, providing a plausible link between intestinal barrier dysfunction and RA pathogenesis (31, 32).

**Figure 2:**
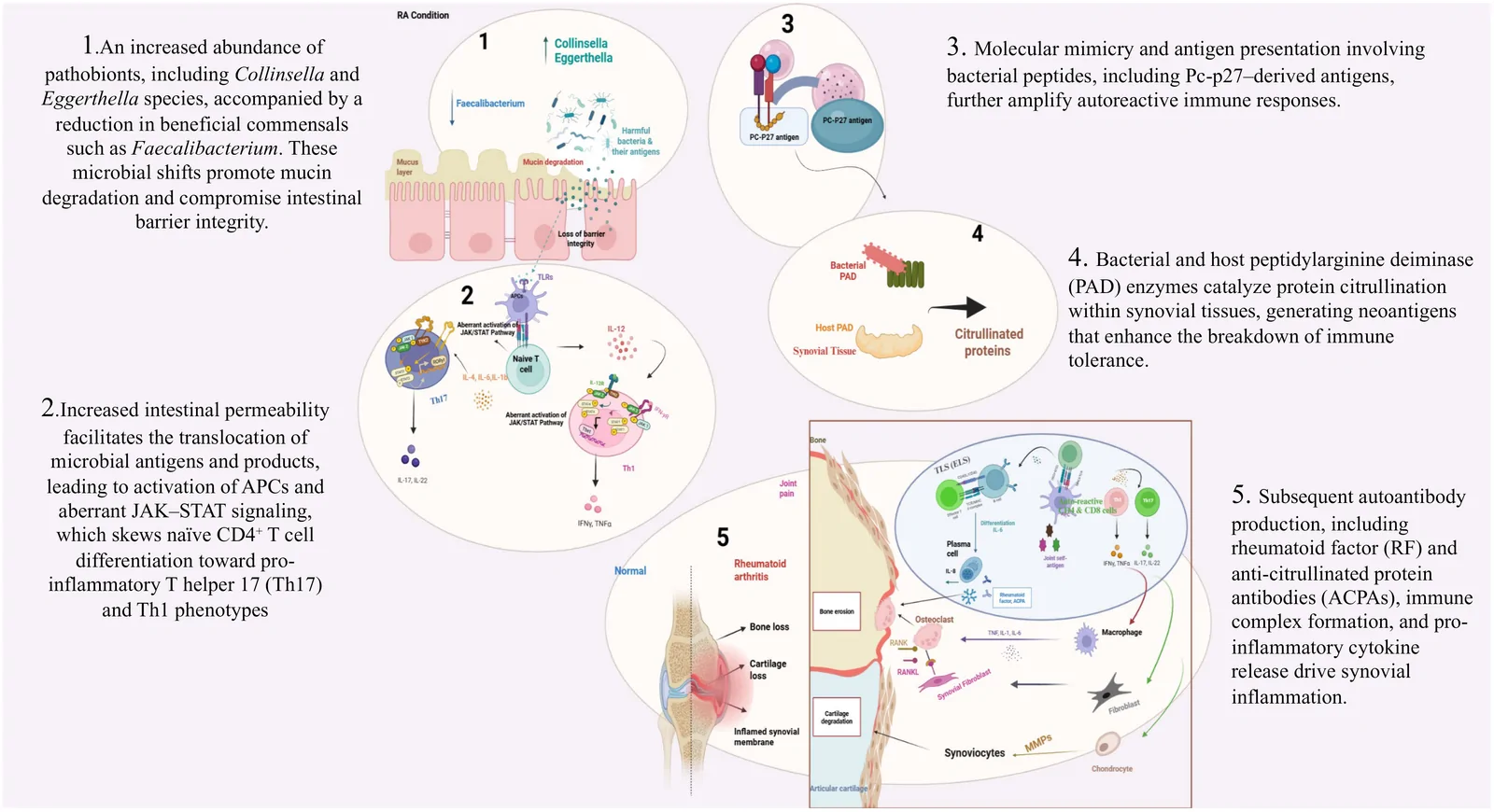
Multistep model linking gut dysbiosis to autoimmunity and joint destruction in rheumatoid arthritis. This schematic illustrates a proposed sequence of events through which alterations in the gut microbiota contribute to the initiation and progression of rheumatoid arthritis (RA). *a* | RA-associated dysbiosis is characterized by an increased abundance of pathobionts, including *Collinsella* and *Eggerthella* species, accompanied by a reduction in beneficial commensals such as *Faecalibacterium*. These microbial shifts promote mucin degradation and compromise intestinal barrier integrity. *b* | Increased intestinal permeability facilitates the translocation of microbial antigens and products, leading to activation of antigen-presenting cells (APCs) and aberrant Janus kinase–signal transducer and activator of transcription (JAK–STAT) signaling, which skews naïve CD4^+^ T cell differentiation toward pro-inflammatory T helper 17 (Th17) and Th1 phenotypes. *c* | Molecular mimicry and antigen presentation involving bacterial peptides, including Pc-p27–derived antigens, further amplify autoreactive immune responses. *d* | Bacterial and host peptidylarginine deiminase (PAD) enzymes catalyze protein citrullination within synovial tissues, generating neoantigens that enhance the breakdown of immune tolerance. *e* | Subsequent autoantibody production, including rheumatoid factor (RF) and anti-citrullinated protein antibodies (ACPAs), immune complex formation, and pro-inflammatory cytokine release drive synovial inflammation, osteoclast activation, cartilage degradation, and bone erosion, culminating in the joint destruction characteristic of established RA. RA, rheumatoid arthritis; APCs, antigen-presenting cells; JAK, Janus kinase; STAT, signal transducer and activator of transcription; Th, T helper; PAD, peptidylarginine deiminase; RF, rheumatoid factor; ACPA, anti-citrullinated protein antibody. Created with BioRender.com.

Beyond disruption of the intestinal barrier, bacterial-derived products may also contribute to RA pathogenesis by generating immunogenic metabolites and promoting protein citrullination. Several bacterial species have been reported to induce citrullination either directly through peptidylarginine deiminase (PAD)- like enzymatic activity or indirectly by activating host pathways that increase PAD expression and activity. PAD enzymes catalyze the conversion of peptidyl-arginine residues to peptidyl-citrulline, generating neoepitopes that can be recognized as autoantigens and stimulate autoantibody production (16). Among these organisms, *Porphyromonas gingivalis*, an oral periodontal pathogen that has also been associated with alterations in gut microbial composition and mucosal immune responses, represents one of the best-characterized microbial contributors to citrullination in RA (33). Its potential role within the broader oral-gut-joint axis is discussed in detail in the following section. The gut-joint axis describes a dynamic and potentially bidirectional communication network through which intestinal microbiota and host immune responses interact in immune-mediated joint diseases, including RA. Current evidence suggests that gut microbial dysbiosis may contribute to RA pathogenesis through multiple interconnected mechanisms, including disruption of intestinal barrier integrity, alterations in the Th17/Treg balance, molecular mimicry, dysregulated microbiota-derived metabolite signaling, and excessive protein citrullination (20, 21, 34). Together, these mechanisms provide a conceptual framework linking intestinal immune perturbations with systemic autoimmunity and joint inflammation (**Figure 2**).

Beyond structural disruption of the intestinal barrier and immune activation, microbiota-derived metabolites have emerged as important regulators of immune homeostasis in RA. Although short-chain fatty acids (SCFAs) remain the best-characterized microbial metabolites, increasing evidence indicates that secondary bile acids (SBAs) and tryptophan-derived metabolites also contribute to host immune regulation (35).

SBAs are generated in the intestine through 7α-dehydroxylation of primary bile acids by anaerobic bacteria, particularly *Clostridium* and *Bacteroides* species (36). Experimental studies suggest that SBAs help preserve intestinal barrier integrity by suppressing the production of pro-inflammatory cytokines, including IL6 and TNFα, reducing intestinal inflammation, and strengthening tight junction integrity (37, 38). SBAs have also been reported to inhibit activation of the NLRP3 inflammasome through the TGR5‐cAMP‐PKA signaling pathway, thereby attenuating intestinal inflammation (39). Furthermore, SBAs promote Treg differentiation while suppressing Th17 cell development through nuclear receptor signaling pathways, including modulation of RORγt, thereby contributing to immune homeostasis (40–43). Emerging evidence also suggest that SBAs influence bone metabolism by enhancing vitamin D absorption and regulating immune–bone interactions. Through suppression of inflammatory cytokines such as IL6 and TNFα and activation of Farnesoid X Receptor (FXR) signaling, SBAs may help limit osteoclastogenesis and bone destruction (44–48).

Tryptophan is another essential amino acid that plays a critical role in maintaining gut–immune homeostasis. Dysregulation of tryptophan metabolism has been associated with several immune-mediated and degenerative diseases, including RA (49, 50). Tryptophan metabolism contributes to intestinal barrier integrity, immune regulation, and protection against bone loss through three principal metabolic pathways: the serotonin, kynurenine, and indole pathways (50, 51). Microbiota-derived indole metabolites, including indole-3-ethanol, indole-3-pyruvate, indole-3-aldehyde, and indole-3-formaldehyde, have been shown to enhance intestinal epithelial barrier integrity by activating the aryl hydrocarbon receptor (AhR), thereby stabilizing tight junction proteins and reducing intestinal permeability under inflammatory conditions (51). These metabolites also stimulate IL-22 production, promoting antimicrobial peptide secretion, supporting colonization by beneficial microorganisms, and limiting pathogen expansion (52). In addition, they can increase the abundance of sugar-fermenting and SCFA-producing bacteria, further reinforcing intestinal homeostasis (52). Deficiency of the microbial sensing gene CARD9 has been linked to impaired microbial tryptophan metabolism, reduced intestinal epithelial proliferation, increased epithelial apoptosis, and disruption of IL-22 signaling (53).

Beyond their effects on barrier integrity, tryptophan metabolites also contribute to regulation of the Treg/Th17 balance, a key determinant of immune homeostasis that is frequently disrupted in autoimmune diseases such as RA. Tryptophan metabolites promote Treg differentiation while suppressing pro-inflammatory Th17 responses, and metabolites such as indole-3-acetic acid further enhance FOXP3 expression while inhibiting the RORγt/STAT3 signaling pathway, thereby promoting immune tolerance (54, 55). Collectively, these findings highlight the immunomodulatory roles of microbiota-derived metabolites in RA.

Current evidence supports three major, non-mutually exclusive mechanisms through which the gut may influence joint inflammation. First, impaired intestinal barrier integrity may permit microbial products to translocate into circulation, promoting systemic inflammation and immune activation (56). Second, molecular mimicry and microbiota-derived metabolites may modulate immune responses and barrier function, thereby influencing joint-specific pathology (20). Third, dysbiosis-derived citrullination and loss of immune tolerance promote the generation of autoantigens and pathogenic adaptive immune responses (20) (**Figure 2**).

### 3.1 Loss of gut barrier integrity

Disruption of intestinal barrier integrity has been proposed as one of the principal mechanisms which gut dysbiosis may contribute to RA pathogenesis. Increased intestinal permeability (“leaky gut”) facilitates the translocation of bacteria and bacterial products, such as LPS, peptidoglycans, and microbial nucleic acids, into the systemic circulation. Notably, elevated circulating zonulin has been detected in ACPA-positive individuals before the onset of clinical arthritis, suggesting that intestinal barrier dysfunction may precede overt disease rather than simply result from chronic inflammation (57). Supporting this concept, a recent metaproteomic study demonstrated that patients with newly diagnosed RA exhibit elevated circulating biomarkers of bacterial translocation together with increased intestinal fatty acid-binding protein (I-FABP), a marker of intestinal epithelial injury, providing direct clinical evidence of impaired intestinal barrier integrity during early RA (58). In a cohort of 125 RA patients, bacterial nucleic acids were detected in both synovial fluid and fecal samples, supporting the concept of microbial translocation in RA (59). Furthermore, 16S rRNA sequencing revealed reduced microbial diversity and a significant expansion of specific taxa, notably *Collinsella*, compared with healthy controls (60) (**Figure 2**).

Increased abundance of *Collinsella* has been linked to reduced expression of the tight junction protein ZO-1, thereby potentially compromising epithelial barrier integrity and facilitating microbial translocation (60). Across multiple independent cohorts, alterations in the abundances of *Collinsella*, *Eggerthella*, and *Faecalibacterium* have frequently been reported in patients with RA; however, the reported microbial findings are not uniform across studies and should be interpreted in the context of differences in study populations, sequencing methodologies, analytical pipelines, and cohort characteristics (12, 34, 60). *Collinsella* and *Eggerthella* are commonly reported to be enriched, whereas *Faecalibacterium*, an anti-inflammatory, butyrate-producing anaerobe, is often reported to be depleted, although the magnitude and direction of these findings vary among studies (12, 34, 60, 61). Reduced abundance of *Faecalibacterium* may contribute to diminished SCFA production, impaired immune regulation, and disease progression, highlighting its potential role in RA pathogenesis and its possible value as a biomarker (34, 61). Furthermore, increased *Collinsella* abundance has been linked to elevated production of the pro-inflammatory cytokine IL-17A and reduced expression of the tight junction protein ZO-1, supporting its potential role in impaired intestinal barrier function (60) (**Table 1**).

**Table 1:** Tight junction protein alterations in rheumatoid arthritis.

Tight junction protein	Role	Findings in RA & inflammatory diseases	Associated diseases & bacteria	References
Occludin	Maintaining barrier integrity and tight junction sealing	Tyrosine phosphorylation of occludin attenuates its interactions with ZO-1, ZO-2, and ZO-3 during inflammation. Lower expression in RA patients. Elevated levels of TNFα mediated internalization of occludin, which elevates gut permeability.	Autoimmune diseases: Rheumatoid Arthritis & MS. Bacteria: Prevotella copri, Collinsella aerofaciens – associated with increased gut permeability and systemic inflammation.	(5, 62, 63)
Claudins	Regulating paracellular transport Backbone of tight junction strands	Levels were significantly lower in RA patients Increased systemic inflammation (e.g., TNF-α, IL-6), disrupts claudin4 via cytokine-mediated signaling.	gastrointestinal diseases: ulcerative colitis and Crohn’s disease Inflammatory bowl disease. Bacteria Lachnospiraceae Veillonella Autoimmune diseases: Rheumatoid Arthritis	(5, 64–66)
ZO-1	Anchors transmembrane tight junction proteins to actin cytoskeleton.	Downregulation of ZO-1 expression in RA	Rheumatoid arthritis. Bacteria: Collinsella aerofaciens	(60)

### 3.2 Molecular mimicry

Molecular mimicry represents another mechanism through which gut dysbiosis may promote autoimmunity. In this process, microbial antigens share structural similarity with host proteins, leading to cross-reactive immune responses that contribute to the breakdown of self-tolerance (67). *Prevotella copri* has been reported to be more abundant in individuals with RA than in healthy controls, including discordant twin studies (68). A 27-kDa *P. copri* protein (Pc-p27) has been shown to induce autoimmunity and exacerbate joint inflammation. Importantly, antibody responses against Pc-p27 correlate positively with anti-citrullinated protein antibody (ACPA) levels and have been detected exclusively in patients with RA (69) (**Figure 2**).

Despite the considerable interest in *P. copri*, microbiome signatures associated with RA remain highly heterogeneous across studies. Although several cohorts have reported an increased abundance of *P. copri* (69, 70), particularly in treatment-naïve patients or those with early RA, subsequent investigations have produced inconsistent findings, with several cohorts failing to replicate this association (71, 72). Increasing evidence suggests that these discrepancies are likely attributable to strain-level differences within *P. copri*, disease stage, and methodological and population heterogeneity than to the mere presence or absence of the species.

Methodological differences likely contribute to these inconsistencies. Differences in analytical methodologies, including DNA extraction procedures, sequencing platforms (16S rRNA gene sequencing vs. shotgun metagenomic sequencing), and bioinformatic pipelines, contribute to inconsistent microbiome findings across RA cohorts (12). Moreover, microbiome sequencing data are inherently compositional because they represent relative rather than absolute microbial abundances. Consequently, differences in normalization strategies, log-ratio transformations, and statistical methods used to account for compositionality can influence the identification of differentially abundant taxa and contribute to discrepancies among studies (73). Therefore, multiple methodological and biological factors must be carefully considered when interpreting cross-study comparisons. Recent literature further underscores that gut microbial profiles vary substantially across cohorts due to differences in geographic location, dietary habits, host characteristics, and study methodologies (12, 74–77). For example, differences in Prevotella abundance have been reported between Western and Asian RA cohorts, while inconsistencies in the reported abundances of higher-level taxa, including *Actinobacteria*, are frequently observed (74–77). In addition, pharmacologic treatments, including tumor necrosis factor (TNF) inhibitors and sulfasalazine, can further reshape gut microbial communities (76, 77). Therefore, establishing a causal role for the gut microbiome in RA will require study designs that extend beyond cross-sectional observations. Longitudinal studies tracking microbiome dynamics in individuals at increased risk of RA, together with microbiota transplantation experiments in germ-free animal models, may provide critical insight into the temporal relationship between gut dysbiosis and disease onset and help clarify causal mechanisms (75, 76).

Additionally, certain bacterial peptides can mimic self-epitopes. For example, bacterial L-asparaginase (L-ASNase) exhibits structural similarity to a type II collagen T-cell epitope. Presentation of this bacterial peptide by antigen-presenting cells (APCs) via HLA-DRB1 may activate autoreactive T cells that subsequently target joint collagen, thereby promoting synovial inflammation (33).

### 3.3 Citrullination and loss of immune tolerance

RA is strongly associated with specific HLA class II alleles, particularly the HLA-DRB1 variants encoding the shared epitope (SE). A central pathogenic event in RA is protein citrullination, a post-translational modification that contributes to loss of immune tolerance. Citrullination is catalyzed by PAD, calcium-dependent enzymes that convert positively charged arginine residues into neutral citrulline. In genetically susceptible individuals, SE-containing HLA-DRB molecules exhibit increased affinity for citrullinated peptides, thereby enhancing their presentation to autoreactive T cells. These T cells promote pathogenic immune responses and provide help to auto-reactive B cells, leading to production of ACPA and IgA rheumatoid factor (RF), often years before the onset of RA (78, 79).

Multiple bacterial species may contribute to excessive citrullination either directly, through bacterial PAD-like activity, or indirectly by increasing substrate availability or inducing host PAD expression. Chen et al. (2016) reported elevated citrulline levels associated with increased abundance of *Eggerthella Lenta*, a bacterium capable of metabolizing ornithine and releasing citrulline as a by-product, thereby potentially fueling ACPA responses (60).

Although the oral-gut-joint axis remains an evolving concept rather than a fully established pathogenic pathway, accumulating evidence supports the existence of a mucosal network linking the oral cavity, gut, and joints in RA (80–82). Periodontitis has been proposed to contribute to RA by serving as a source of chronic oral inflammation that promotes systemic autoimmunity (83). Swallowed oral microorganisms may influence gut microbial composition, while persistent oral mucosal inflammation has been suggested to contribute to systemic immune activation and dysregulation (83). Together, these observations support a potential oral–gut–joint connection, although the precise mechanisms remain to be fully elucidated. Consistent with this concept, periodontitis has been associated with the onset and progression of several systemic inflammatory diseases, including RA (83). Notably, the oral pathogen *Porphyromonas gingivalis* has been detected in the synovial fluid of patients with RA and uniquely expresses a bacterial peptidylarginine deiminase (PAD) enzyme capable of citrullinating host proteins. This process has been linked to enhanced production of pro-inflammatory cytokines, including IL-6 and IL-17, thereby reinforcing autoimmune inflammation and joint pathology (84). Supporting the involvement of the intestinal mucosa in RA, Bennike et al. (2017) identified multiple citrullinated peptides in colonic tissue from patients with RA, suggesting local citrullinated antigen generation in the setting of intestinal barrier dysfunction (85).

Within the joint microenvironment, PAD activity contributes to the accumulation of pro-inflammatory citrullinated proteins, including citrullinated fibrin. Dual-modified proteins bearing both citrullination and malondialdehyde-acetaldehyde adducts (MAA) elicit particularly strong immune responses in RA (86, 87). Rheumatoid arthritis synovial fibroblasts (RASFs) respond robustly to PAD-citrullinated fibrin by producing IL-6 and IL-8, thereby potentially amplifying local inflammation. In addition, autoantigenic citrullinated proteins identified in synovial fluid include collagen II, fibronectin, and vimentin (86). Activation of macrophages by dual-modified proteins induces the secretion of multiple growth factors, including platelet-derived growth factor (PDGF), which may further promote the aggressive phenotype and pathogenic differentiation of RASFs (87). Collectively, these findings suggest that excessive citrullination, whether arising from host or microbial mechanisms, may contribute to synovial inflammation and disease progression in RA. Furthermore, sustained citrullination and inflammatory signaling may favor Th17 expansion while impairing Treg stability, thereby perpetuating chronic autoimmune inflammation.

## 4 Breakdown of immune tolerance: Th17/Treg imbalance

**Figure 3:**
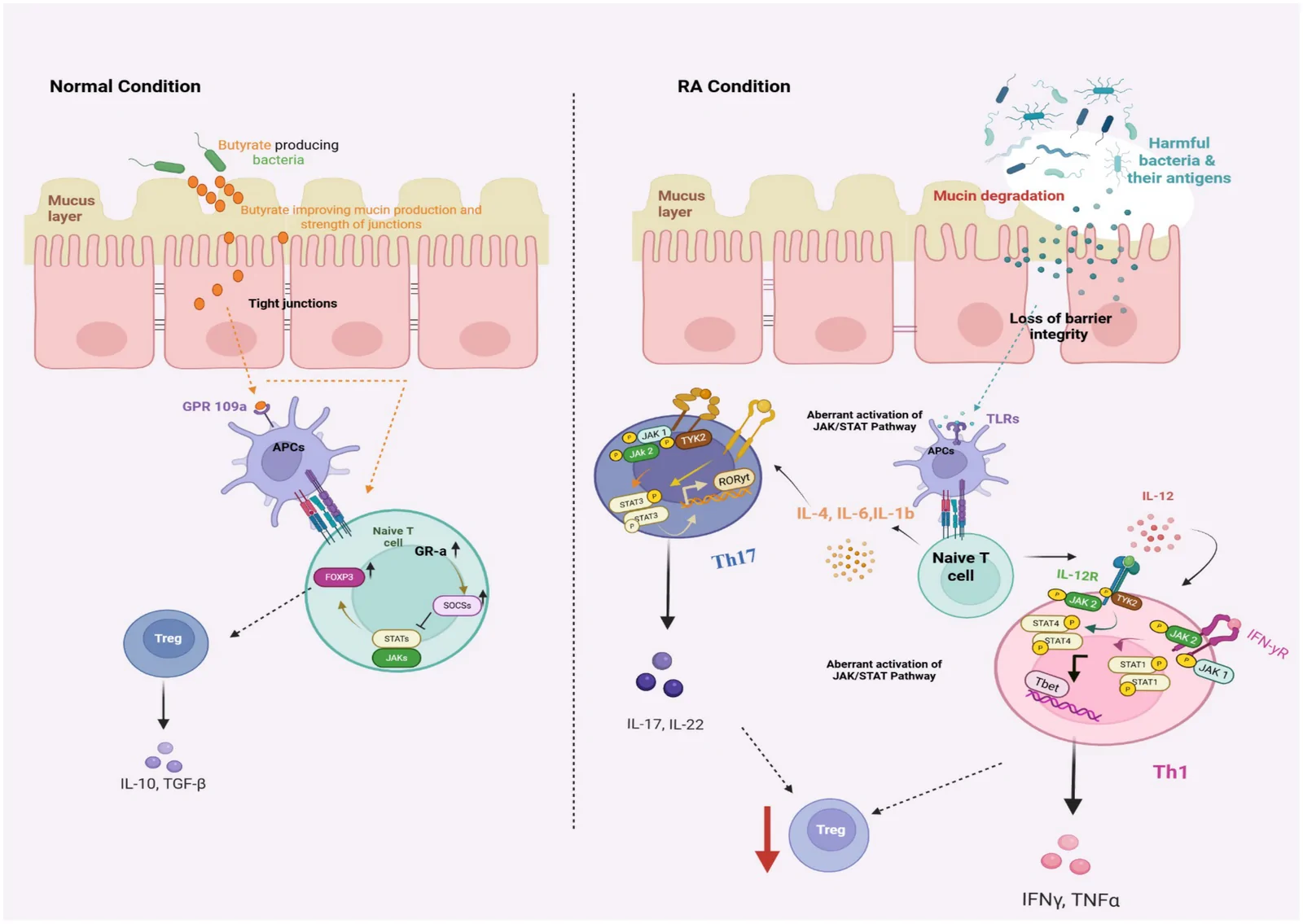
Intestinal immune homeostasis in health and immune dysregulation in rheumatoid arthritis. This schematic contrasts intestinal immune homeostasis under healthy conditions (left) with immune dysregulation observed in rheumatoid arthritis (RA) (right). Under homeostatic conditions, commensal bacteria produce short-chain fatty acids (SCFAs), particularly butyrate, which enhance mucin production, reinforce epithelial tight junctions, and preserve intestinal barrier integrity. Butyrate signaling through G protein-coupled receptor 109A (GPR109A) on antigen-presenting cells (APCs) promotes regulatory T cell (Treg) differentiation and the production of anti-inflammatory cytokines, including interleukin-10 (IL-10) and transforming growth factor-β (TGF-β). In RA, gut dysbiosis characterized by an increased abundance of pathobionts leads to mucin degradation and loss of epithelial barrier integrity. Translocation of microbial products activates Toll-like receptors (TLRs) on APCs, resulting in aberrant activation of Janus kinase–signal transducer and activator of transcription (JAK–STAT) signaling pathways. This inflammatory milieu skews naïve CD4^+^ T cell differentiation toward pro-inflammatory T helper 17 (Th17) and Th1 phenotypes, which secrete IL-17, IL-22, interferon-γ (IFN-γ), and tumor necrosis factor-α (TNF-α), thereby promoting systemic inflammation and joint pathology. RA, rheumatoid arthritis; SCFAs, short-chain fatty acids; APCs, antigen-presenting cells; Treg, regulatory T cell; TLRs, Toll-like receptors; JAK, Janus kinase; STAT, signal transducer and activator of transcription; Th, T helper. Created with BioRender.com.

Th17 and Treg cells are two critical CD4^+^ T-cell subsets with opposing immunological functions that together maintain immune homeostasis. Tregs are anti-inflammatory cells that suppress excessive immune responses and maintain self-tolerance, whereas Th17 cells are pro-inflammatory and are primarily characterized by the production of IL-17 (88, 89). An imbalance favoring Th17 cells over Tregs has been consistently observed in a wide range of pathological conditions, including autoimmune diseases, metabolic disorders, and cancer, and is considered a hallmark of immune dysregulation (89, 90) (**Figure 3**).

Accumulating evidence suggests that the gut microbiota plays an important role in regulating the differentiation and function of Th17 and Treg cells (2, 91, 92). Specific commensal bacterial strains promote the generation of microbiota-induced Tregs, which help protect the intestinal mucosa from excessive immune activation and maintain tolerance to dietary antigens. In contrast, Th17 cells are essential for host defense against extracellular pathogens but may become pathogenic when dysregulated (2, 91, 92). Consequently, alterations in microbial composition have the potential to influense T-cell polarization and immune homeostasis.

During the early phase of RA, gut-derived immune signals have been proposed to contribute to systemic immune activation. In particular, gut-associated Th17 cells have been suggested as a potential mechanistic link between intestinal dysbiosis and RA by facilitating immune cell trafficking from mucosal sites to the joints (93). Experimental studies using CIA mouse models and microbiota transfer experiments with samples from RA patients have demonstrated increased Th17 cell frequencies in serum and synovial fluid relative to Tregs (32). Although these findings support a role for gut microbiota in modulating adaptive immunity, further longitudinal human studies are needed to establish the temporal and causal relationships between dysbiosis, Th17 expansion, and RA development.

Aberrant activation of innate immunity, together with dysregulated T- and B- cell responses and pathogenic autoantibody production, further favors Th17 differentiation while impairing Treg development and function (89). This immune imbalance is associated with elevated levels of pro-inflammatory cytokines, including IL-17, IL-12, IL-23, and type I interferons, accompanied by reduced production of anti-inflammatory mediators such as IL-10 and transforming growth factor-β (TGF-β) (19). The resulting predominance of Th17 cells, together with numerical and functional deficiencies in Tregs, is associated with cartilage degradation, osteoclast differentiation, joint destruction, and progressive bone loss in RA (94).

In established RA, the inflammatory cytokine milieu further compromises Treg stability and suppressive capacity. Elevated levels of IL-6, IL-1β, and TNF-α impair Treg function by downregulating the transcription factor FOXP3 and its downstream target CTLA-4, a key mediator of Treg suppressive activity (95). Since FOXP3 directly regulates CTLA-4 transcription, reduced FOXP3 expression contributes to Treg destabilization and may promote the emergence of so-called “ex-Treg” cells with diminished suppresive activity within the synovial microenvironment (95, 96). Moreover, IL-6 promotes Th17 differentiation and has alos been reported to induce inhibitory receptors, including PD-1 and LAG-3, thereby contributing to Treg dysfunction and exhaustion (97, 98).

At the molecular level, IL-6 is a key regulator of Th17 differentiation through activation of STAT3 signaling pathway. Following IL-6 receptor engagement, STAT3 becomes phosphorylated and translocates to the nucleus, where it induces expression of the Th17-lineage–defining transcription factor RORγt (99). Concurrently, IL-6 destabilizes Tregs by impairing FOXP3 expression at both transcriptional and post-translational levels. Transcriptionally, IL-6 promotes DNA methylation at CpG islands within FOXP3 enhancer regions, whereas post-translationally it induces phosphorylation of FOXP3 at serine 422, reducing its chromatin-binding capacity and transcriptional activity (100). Collectively, these mechanisms provide a plausible molecular framework through which dysbiosis-associated inflammatory signaling may disrupt Th17/Treg balance, contributing to breakdown of immune tolerance and persistent inflammation in RA.

### 4.1 Immune cell trafficking from mucosa to joint damage

Multi-step interactions among microbial imprinting, epithelial barrier disruption, and shared homing pathways have been proposed to facilitate gut-to-joint trafficking of immune cells and contribute to RA pathogenesis.

Following disruption of the intestinal barrier, microbial-associated molecular patterns (PAMPs), including LPS, flagellin, and bacterial nucleic acids, may translocate into the systemic circulation, where they engage pattern-recognition receptors, particularly TLR4, on monocytes, macrophages, and DCs (101). Elevated levels of the chemokines CCL22 and CXCL8, which are associated with increased monocyte and neutrophil infiltration and activation within the joints, have been detected in the synovial fluid and serum of patients with arthritis (102). Activation and recruitment of these immune cells may influence the synovial immune response by promoting the production of pro-inflammatory cytokines, including TNF-α, IL-1β, and IL-6 (103). These cytokines are important drivers of Th17 differentiation (104). Furthermore, recruitment and activation of neutrophils within synovial tissue may promote cartilage degradation and bone erosion through the release of matrix-degrading enzymes (105). Collectively, these findings suggest that neutrophil infiltration and M1 macrophage polarization are characteristic features of inflammatory bone disease in RA.

As the most abundant circulating leukocytes, neutrophils constitute a critical component of innate immunity (106). Following recruitment to sites of inflammation, neutrophils undergo degranulation, phagocytosis, and the formation of neutrophil extracellular traps (NETs) (107). NETs are web-like structures composed of DNA, histones, and granule proteins released by activated neutrophils. Beyond their established role in antimicrobial defense, NETs are increasingly recognized as important mediators of inflammation and autoimmunity (108). Several NET-associated molecules, including citrulline peptides, histones, and myeloperoxidase, are released during NET formation and function as autoantigens, supporting a role for NETs in the pathogenesis of autoimmune diseases (109). Increased NET formation has been detected in the inflamed intestinal mucosa and peripheral blood of patients with active inflammatory bowel disease (IBD), where NET abundance correlates with disease severity (110, 111). Elevated circulatory NETs have been reported in patients with sepsis and in placental tissue from spontaneous miscarriages (112, 113). Patients with RA exhibit increased neutrophil accumulation within synovial fluid and vascular membranes compared to healthy individuals (109). The presence of activated neutrophils and NETs formation within affected joints may promote inflammatory cell recruitment and cytokine production, including IL-1β, thereby contributing to persistent synovial inflammation (114).

The majority of intestinal CD4^+^ T cells are memory or effector T cells whose differentiation is shaped by the microbial antigenic composition, niche localization, and metabolic activity of the gut microbiota (115). Antigen presentation by DCs promotes polarization toward inflammatory subsets, including Th17 cells, and may support the generation of autoreactive B cell responses. Importantly, structural similarity between microbial antigens and RA- associated self-epitopes may facilitate molecular mimicry and contribute to the loss of immune tolerance (81).

Within gut-associated lymphoid tissues (GALT)- including Peyer’s patches and mesenteric lymph nodes, DCs imprint activated T cells with gut-homing properties through gut-derived signals such as retinoic acid, inducing the expression of the chemokine receptor CCR9 and the integrin α4β7. Under physiological conditions, engagement of CCR9 with its ligand CCL25 and of α4β7 with mucosal addressin cell adhesion molecule-1 (MAdCAM-1) establishes a tissue-restricted recirculation pathway that directs lymphocytes back to the intestinal mucosa. Under inflammatory conditions, however, ectopic expressions of MAdCAM-1 and gut-associated chemokines has been reported in extra-intestinal tissues, including the synovium, potentially facilitating recruitment of gut-imprinted lymphocytes (116).

**Figure 4:**
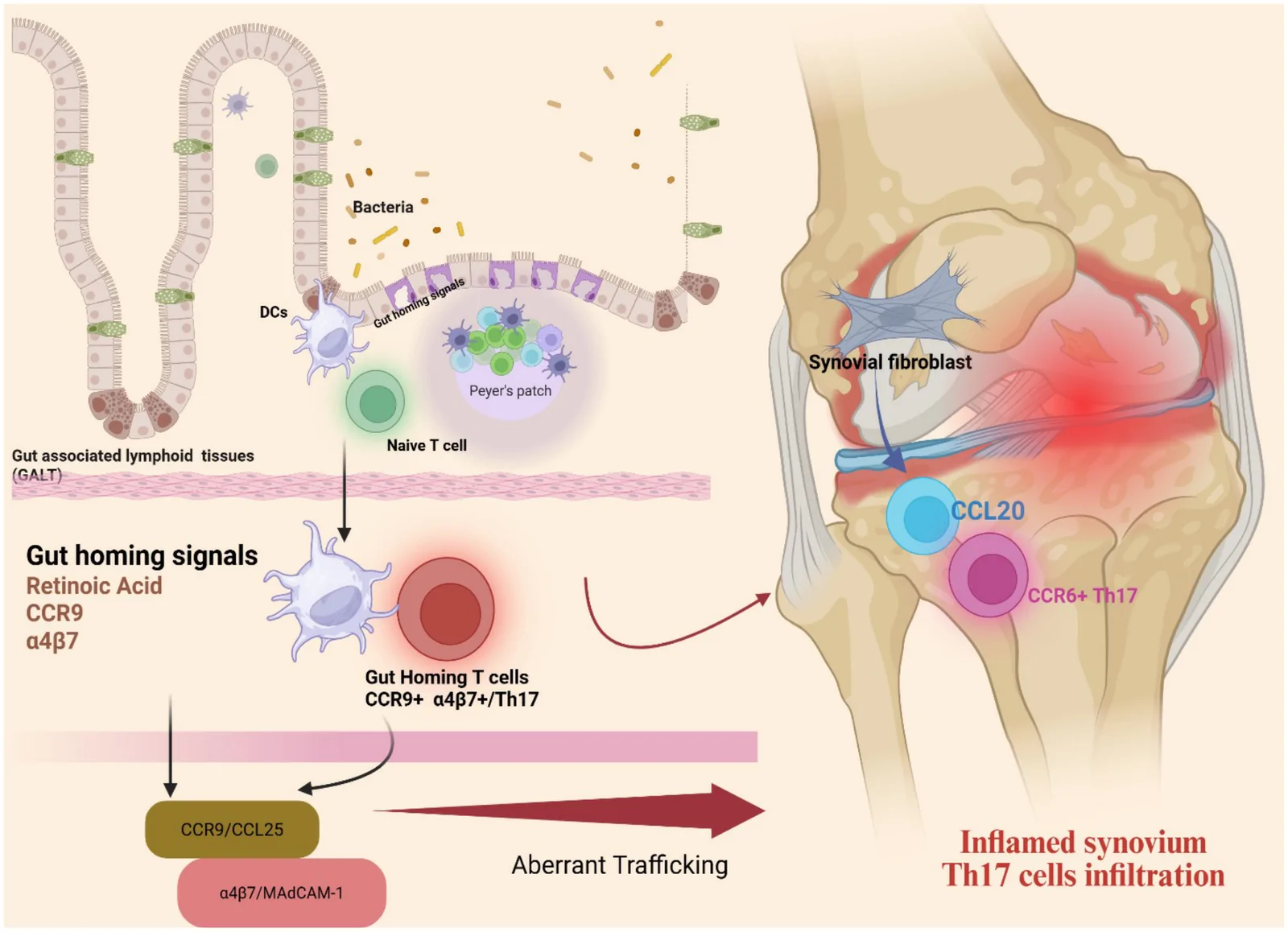
Aberrant trafficking of gut-imprinted Th17 cells to the joints in rheumatoid arthritis. This schematic illustrates how intestinal immune activation contributes to synovial inflammation via dysregulated T-cell trafficking in rheumatoid arthritis (RA). Within the gut-associated lymphoid tissue (GALT), including Peyer’s patches, microbial antigens are sampled and presented by dendritic cells (DCs), leading to the activation of naïve CD4^+^ T cells. Local cues, including retinoic acid, imprint activated T cells with gut-homing properties by inducing expression of the chemokine receptor CCR9 and the integrin α4β7. Under pathological conditions, a subset of CCR9^+^ α4β7^+^ T helper 17 (Th17) cells aberrantly exits the intestinal compartment and migrates to peripheral joints rather than returning to the gut. Within the synovium, synovial fibroblasts and resident immune cells secrete CCL20, promoting the recruitment and retention of CCR6^+^ Th17 cells. The accumulation of Th17 cells in the inflamed synovial tissue amplifies local cytokine production and sustains chronic inflammation, thereby contributing to RA pathogenesis. RA, rheumatoid arthritis; GALT, gut-associated lymphoid tissue; DCs, dendritic cells; Th17, T helper 17 cell. Created with BioRender.com.

In RA, synovial fibroblasts and infiltrating immune cells produce chemokines such as CCL20, generating a chemotactic gradient that selectively recruits CCR6^+^ Th17 cells (116). In addition, increased expression of CCL25 and aberrant expression of MAdCAM-1 within inflamed joints may further promote adhesion and retention of gut-imprinted lymphocytes. Together, these mechanisms may redirect gut-homing T cells—particularly Th17 cells—to the synovium (**Figure 4**), where they may amplify local inflammation and contribute to RA pathogenesis (116–119).

Gut-homing molecules, including integrins and chemokine receptors, play important role in directing intestinal lymphocyte trafficking. Notably, these molecules are aberrantly expressed in the synovium of patients with inflammatory arthritis, supporting the concept that immune cells imprinted within the gut microenvironment may migrate to and accumulate in the joints (120, 121). Integrins such as α4β7 and αEβ7, which normally mediate lymphocyte homing to the intestinal mucosa, have been detected on T cells within synovial tissue and fluid, suggesting preservation of a gut-homing imprint that becomes misdirected under inflammatory conditions (120, 121).

In parallel, chemokine pathways involved in intestinal lymphocyte trafficking appear to be functionally reconstituted within the inflamed synovium. Monocytes and macrophages from patients with RA express the gut-associated chemokine receptor CCR9, and its ligand CCL25,normally restricted to the small intestine, may promote their differentiation and activation, establishing a chemotactic pathway analogous to that operating in intestinal tissues (121). Consequently, this aberrant CCR9–CCL25 axis may facilitate recruitment and retention of mucosa-imprinted immune cells within the joints, thereby contributing to local inflammation.

Within the synovial compartment, αEβ7-expressing T cell populations, including Th17, T follicular helper (Tfh), and γδ T cells, have been identified, further supporting the potential contribution of gut-derived immune populations to joint pathology (117). Complementary evidence is provided by studies of axial spondyloarthritis (axSpA), in which mucosal-associated invariant T (MAIT) cells express high levels of gut-homing receptors, including CCR9 and integrin CD49d, following infiltration of synovial tissue (122). Importantly, TNF blockade reduces both the activation status and functional capacity of synovial MAIT cells, suggesting that inflammatory cues help sustain their pathogenic activity within joints (**Table 2**) (122).

**Table 2:** Expression of gut-homing receptors in rheumatoid arthritis synovium.

Cell type/subset	Gut homing/Trafficking markers	Evidence for presence in synovium	Implication for Gut–Joint Axis	References
Conventional T cells (αβ T cells)	α4β7, αEβ7	High β7 integrin (α4β7, αEβ7) expression on synovial T cells.	Gut primed T cells expressing gut homing integrin migrate to synovium	(120)
Mucosal-Associated Invariant T (MAIT) cells	CD49d (α4 integrin) CCR9 (chemokine receptor)	High frequency of CD3+CD8+ MAIT expressing CCR9 and CD49d in axSpA patients	Showing that gut associated MAIT cells detected in synovial biopsy.	(122)
Tc17 T cells	β7 integrin (especially αEβ7), CXCR6	Tc17 cells expressing CXCR6 and β7 integrin have been identified in the synovial fluid of patients with psoriatic arthritis and juvenile idiopathic arthritis. In rheumatoid arthritis, Tc17 cells have been reported in peripheral blood, synovial fluid, and synovial tissue and correlate with disease activity; however, expression of CXCR6 and gut-homing markers has not been specifically demonstrated.	Supports the concept that gut-associated cytotoxic T-cell subsets may contribute to joint inflammation across inflammatory arthritides; however, direct evidence for gut-homing Tc17 cells in RA remains limited.	(123, 124)

Persistent recruitment and retention of immune cells within the inflamed synovium may also contribute to the formation of tertiary lymphoid structures (TLS), ectopic lymphoid aggregates that resemble secondary lymphoid organs (125). These structures contain organized T-cell and B-cell zones, dendritic cells, follicular dendritic cells, high endothelial venules, and plasma cells, thereby creating a local microenvironment that supports antigen presentation, B-cell maturation, germinal center-like reactions, and local autoantibody production (125). Consequently, TLS may sustain chronic synovial inflammation by perpetuating adaptive immune responses independently of secondary lymphoid organs. The inset in **Figure 2** illustrates the proposed organization of TLS within the inflamed synovium and highlights their potential role in linking mucosal immune activation with persistent joint inflammation.

Collectively, these findings support a model in which gut-imprinted immune cells may be redirected to the synovium through shared homing pathways, where they contribute to persistent cytokine production, TLS formation, macrophage activation, and tissue-destructive inflammation. Although accumulating experimental and clinical evidence supports this gut–joint trafficking paradigm, additional longitudinal human studies are needed to clarify its temporal sequence and causal contribution to RA pathogenesis.

### 4.2 Therapeutic strategies addressing gut dysbiosis and microbial metabolites in rheumatoid arthritis

Current management of RA follows a stepwise, mechanism-driven therapeutic approach aimed at controlling inflammation, preventing structural joint damage, and maintaining long-term remission. Beyond their established immunomodulatory effects, DMARDs also interact with the gut microbiome. Clinical and metagenomic studies have shown that conventional DMARD therapy, particularly methotrexate, is associated with partial restoration of disease-associated gut microbial dysbiosis, while pretreatment gut microbiome has also been linked to subsequent treatment response (81, 126). Sulfasalazine may also influence gut microbial composition through its dependence on bacterial metabolism for activation and its intrinsic antibacterial activity; however, evidence supporting consistent modulation of the gut microbiome in patients with RA remains limited and heterogeneous (34). Collectively, these findings suggest a bidirectional relationship in which RA therapies influence the gut microbiota, and microbial composition may, in turn, affect therapeutic efficacy. In contrast, current evidence indicates that biologic DMARDs, including TNF inhibitors, abatacept, and tocilizumab, exert relatively limited direct effects on gut microbial composition, with observed microbial changes likely occurring secondary to suppression of inflammation and restoration of mucosal immune homeostasis. Although TNF inhibitors have been associated with modest improvements in microbial diversity in some studies, these findings remain preliminary and require further validation (77).

Gut dysbiosis in patients with RA is characterized by the loss of beneficial microbial taxa and a concomitant reduction in bioactive microbial metabolites, including amino acids, SCFAs, secondary bile acids, and tryptophan metabolites (127). The principal SCFAs produced in the human gut, acetate, propionate, and butyrate, together with lesser-abundant SCFAs such as pentanoate, hexanoate, and heptanoate, play critical roles in maintaining intestinal barrier integrity and immune homeostasis (128). In RA, dysbiosis has been associated with depletion of butyrate-producing bacteria together with enrichment of butyrate-consuming species, resulting in reduced bioavailable butyrate (129). This metabolic imbalance has been associated with increased autoantibody production, bone erosion, and greater disease severity (129). Importantly, evidence from individuals at risk of RA suggests that reduced butyrate may precede the onset of clinically apparent disease rather than merely reflecting established inflammation. In a prospective cohort of ACPA-positive individuals, lower serum butyrate concentrations were significantly associated with progression to clinical arthritis, supporting a potential role for microbial metabolites in the earliest stages of RA development (130).

Mechanistic studies using cell culture systems and animal models further support a protective role for butyrate in inflammatory arthritis. In CIA and AIA models, butyrate concentrations are markedly reduced during the acute phase of disease compared with pre-arthritic states. Therapeutic butyrate supplementation attenuates intestinal inflammation, restores epithelial barrier function, reduces serum zonulin levels, and ameliorates arthritis severity (5). Although these findings are encouraging, confirmation in human studies remains limited.

SCFAs exert their anti-inflammatory effects through two principal mechanisms: (i) epigenetic regulation via inhibition of histone deacetylases (HDACs), and (ii) receptor-mediated signaling through G protein–coupled receptors (GPCRs), including FFAR2 (GPR43), FFAR3 (GPR41), and GPR109A (HCAR2), which are expressed on epithelial and immune cells (131). Through these pathways, SCFAs suppress pro-inflammatory cytokines such as IL-6 while enhancing anti-inflammatory mediators including IL-10, thereby promoting immune tolerance (131).

Importantly, SCFA signaling may have therapeutic implications beyond immune modulation. Steroid hyporesponsiveness, a major challenge in chronic inflammatory diseases, has been associated with altered glucocorticoid receptor (GR) signaling and reduced HDAC2 activity. Glucocorticoids exert anti-inflammatory effects by binding cytosolic GRs, promoting nuclear translocation, and subsequently activating glucocorticoid response elements (GREs) or repressing inflammatory transcription factors such as NF-κB through HDAC2 recruitment (132). Although SCFAs are class I/II HDAC inhibitors, they may indirectly preserve HDAC2 function under inflammatory conditions by reducing oxidative stress and improving GR signaling. In addition, activation of FFAR2, FFAR3, and GPR109A by SCFAs modulates cytokine production by suppressing IL-6 while enhancing IL-10 and promoting FOXP3 expression, thereby supporting T-cell differentiation and immune tolerance (131, 133).

Current management of RA follows a stepwise, mechanism-based approach aimed at controlling inflammation, preventing structural joint damage, and achieving sustained disease remission. Complementing conventional therapies, restoration of microbial homeostasis and enhancement of SCFA-producing bacteria through microbiota-directed interventions represents have emerged as promising adjunctive strategies. Preclinical and early clinical studies suggest that probiotics, prebiotics, dietary fiber supplementation, and FMT may reshape gut microbial composition, increase SCFA availability, and modulate immune responses relevant to RA pathogenesis (**Table 3**) (145).

**Table 3:** Therapeutic strategies targeting the gut microbiota in rheumatoid arthritis.

Category	Agent	Gut microbiota modulation	Anti-RA mechanism	Reference
Probiotic	*Lactobacillus* spp.	Restores microbial balance, increases SCFA-producing bacteria and reduction in dysbiosis	Ø Reduces proinflammatory cytokines Ø Less joint swelling Ø Lower arthritis scores Ø Less bone destruction Ø A reduction in white blood cell count and TNF-α and IL-6 plasma levels	(134, 135)
Probiotic	*Multi-strain probiotic formulations*	Improve gut microbial diversity and intestinal barrier function	Ø Significant decrease in IL-1β levels Ø Attenuate systemic inflammation and disease activity	(136)
Prebiotics	Prebiotic dietary fibers	Significant increase in the production of acetate and butyrate in the cecum Higher total amount of SCFAs Enhanced the integrity of the intestinal barrier by regulating the proteins ZO-1 and Claudin-1 in the colon. Reduced the expression of inflammatory factors IL-6 and TNF-α in colon tissue, and increased the expression of IL-10	Ø Modulation of the Treg/Th17 immune balance. Ø Lower levels of pro-inflammatory cytokine IL-17	(137–139)
Natural Products				
Polyphenol	Resveratrol	Enriches beneficial microbiota and microbial metabolites. Also Improves gut microbiota composition	Ø Combined anti-inflammatory, immunomodulatory, and antioxidant activities. Ø Reduces pro-inflammatory cytokines. Ø promotes Treg-mediated immune tolerance	(140)
Polyphenol	Curcumin	Ameliorate gut dysbiosis by reshaping gut microbial composition. Promoting beneficial microbial metabolites. Enhancing intestinal barrier function and suppressing inflammation.	Ø Significant reductions in C-reactive protein (CRP) Ø Decrease inflammation.	(141, 142)
Flavonoid	Quercetin	Increases beneficial commensals and reduces pathogenic bacteria	Ø Suppressing immune activation linked to atherosclerosis progression.	(143)
Alkaloid	Berberine	Significantly remodels the intestinal microbiota by increasing beneficial bacteria such as *Akkermansia* and SCFA-producing taxa while reducing pathogenic microbes linked to dysbiosis.	Ø Aalleviates arthritis severity by suppressing inflammatory signaling pathways including NF-κB Ø Regulating immune responses, and correcting metabolic disturbances associated with RA	(144)
Standard RA pharmacotherapy				
Conventional DMARD	Methotrexate	Associated with partial restoration of disease-associated gut dysbiosis. Baseline gut microbiota composition has also been associated with subsequent treatment response.	Ø Suppresses immune activation and synovial inflammation by inhibiting lymphocyte proliferation and reducing pro-inflammatory cytokine production.	(81, 126)
Conventional DMARD	Sulfasalazine	Requires bacterial metabolism for activation and may alter gut microbial composition through its antibacterial activity; however, direct effects on the RA gut microbiome remain limited and inconsistent.	Ø Suppresses inflammatory responses through multiple immunomodulatory mechanisms, including inhibition of prostaglandin synthesis, leukocyte function, and pro-inflammatory cytokine production.	(34, 77)
Biologic DMARD	TNF inhibitors	Limited direct effects on gut microbial composition; may modestly improve microbial diversity indirectly through suppression of inflammation and restoration of mucosal homeostasis.	Ø Neutralizes TNF-α and suppresses inflammatory signaling.	(77)
Biologic DMARD	Abatacept/ Tocilizumab	Current evidence suggests minimal direct microbiome modulation; observed changes are likely secondary to improved disease control.	Ø Inhibits T-cell co-stimulation (abatacept) or IL-6 signaling (tocilizumab).	(77)

Experimental studies have demonstrated that SCFAs, particularly butyrate, regulate DC differentiation, antigen presentation, and cytokine production. Butyrate interferes with monocyte-derived DC maturation and function and modulate IL-23 production, a cytokine essential for Th17 cells, thereby providing a mechanistic link between microbial metabolites and adaptive immune regulation (146).

Probiotic supplementation, particularly with *Lactobacillus* and *Bifidobacterium* species, has been shown in CIA and other murine models of RA to reduce joint inflammation, limit cartilage damage, and restore microbial balance by suppressing pathogenic taxa and modulating host immune responses. Clinical studies have also suggested potential benefits of probiotic supplementation, including reductions in disease activity scores and inflammatory biomarkers in some patient cohorts; however, these findings remain heterogeneous because of differences in probiotic strains, treatment duration, patient characteristics, concomitant medications, and study design. Therefore, although probiotics represent a promising adjunctive strategy, larger well-designed randomized controlled trials are required to establish their clinical efficacy in RA (147, 148).

Prebiotics similarly promote the selective expansion of beneficial commensal bacteria and increase SCFA production, thereby supporting intestinal barrier integrity and immune homeostasis. Among these, inulin, a fructan-type polysaccharide, has demonstrated anti-inflammatory effects in experimental models of RA, largely through increased butyrate production and attenuation of systemic inflammation (137, 149). Although some clinical studies have reported reductions in inflammatory biomarkers, including C-reactive protein (CRP) and TNF-α, following inulin supplementation, the available evidence remains limited and heterogeneous. Additional well-powered randomized controlled trials are needed to define their therapeutic role in RA (150).

Beyond its immunomodulatory effects, inulin has been shown to preserve intestinal barrier integrity by enhancing the expression of tight junction proteins, including ZO-1, occludin, and claudins, reducing intestinal permeability, and modulating gut microbial composition toward increased microbial diversity and a more balanced microbial community. In addition, inulin suppresses intestinal pro-inflammatory cytokines while increasing IL-10 expression, further supporting intestinal immune homeostasis. Although these barrier-protective effects have been demonstrated in experimental models of intestinal dysfunction rather than RA, they provide mechanistic evidence supporting the potential role of inulin in restoring gut barrier function. Furthermore, inulin supplementation has been associated with increased fecal and serum SCFA concentrations, particularly butyrate, in patients with RA, supporting its role in microbiota-derived metabolic regulation (137, 149).

Additionally, FMT represents a more direct approach to restoring microbial diversity and function. In preclinical studies, FMT from healthy donors inhibited osteoclastogenesis and bone loss in ovariectomy-induced mouse models while enhancing the expression of the tight junction protein ZO-1, suggesting a link between restoration of microbial homeostasis, intestinal barrier integrity, and skeletal health (151). Although early-phase clinical studies indicate improvements in disease activity following FMT in autoimmune conditions, including RA, clinical translation remains limited by challenges in donor selection, procedural standardization, long-term safety, and regulatory considerations (152).

Despite substantial progress in understanding the relationship between gut microbiota-derived metabolites and immune regulation in RA, the precise molecular mechanisms underlying these interactions remain incompletely understood. Emerging technologies, including single cell transcriptomics, metabolomics, personalized microbiota profiling, artificial intelligence-assisted bioinformatics, and multi-omics approaches, together with well-designed longitudinal studies, are expected to improve patient stratification, identify disease- associated microbial signatures, and accelerate the translation of microbiome research into precision therapeutic strategies for RA (153–155).

### 4.3 Limitations in clinical evidence for microbiota-based therapies in rheumatoid arthritis

Although microbiota-based therapies, such as FMT, are supported by encouraging mechanistic and preclinical evidence linking the gut microbiota to immune-mediated joint inflammation, their clinical efficacy in RA remains uncertain. A single case report described clinical improvement in a 20-year-old woman with RA following FMT treatment from a healthy 8-year-old donor (156). However, evidence from isolated case reports, animal studies, and small exploratory controlled studies are insufficient to establish the efficacy or safety of FMT in RA, highlighting the need for well-designed randomized controlled trials (157).

Similarly, although probiotics and prebiotics have attracted considerable interest as adjunctive therapeutic approaches, current clinical evidence remains limited and inconsistent. Several clinical trials have reported improvements in inflammatory biomarkers and disease activity; however, these findings have not been consistently replicated, and other studies have demonstrated minimal or no significant clinical benefit (147). Furthermore, a systematic evaluation found no significant difference in disease activity between participants receiving probiotics and control groups, although interpretation of these findings is limited by small sample size, short intervention periods, limited follow-up, and the inclusion of patients receiving concurrent RA therapies rather than treatment-naïve populations (134). Because DMARDs and biologic therapies can independently alter both gut microbiota composition and disease activity, concomitant medication use represents an important confounding factor in microbiome-based intervention studies. Consequently, larger, adequately powered randomized controlled trials are required to determine the therapeutic value of probiotics in RA (158).

Likewise, clinical evidence supporting FMT in RA remains limited to small exploratory studies, with no large randomized controlled trials completed to date. Although preclinical studies demonstrate promising immunoregulatory effects, careful evaluation of the risks and benefits of FMT remains essential. Continued refinement of donor screening protocol is necessary to minimize the transmission of microorganisms that could lead to adverse infectious events (159). Donor selection requires comprehensive medical history assessment with rigorous screening for infectious diseases to ensure both safety and efficacy (160). In addition, FMT may inadvertently transfer microorganisms associated with other chronic diseases, including cardiovascular and neurologic disorders, underscoring the need for continued long-term safety evaluation (161).

## 5 Conclusion remarks

In conclusion, accumulating evidence suggests that gut dysbiosis and mucosal immune dysfunction may play important roles in the pathogenesis of RA, although these mechanisms should be considered within the broader context of the disease’s multifactorial etiology. The four-layer intestinal barrier framework proposed in this review provides an integrative conceptual model describing how microbial dysbiosis, impaired intestinal alkaline phosphatase (IAP) activity, mucus depletion, epithelial barrier disruption, and alterations in antimicrobial defenses may contribute to immune activation, systemic inflammation, and joint pathology. By organizing current evidence within this sequential framework, this review extends beyond summarizing individual pathogenic mechanisms and offers a structured perspective that may facilitate the identification of disease endotypes and inform the development of barrier-targeted therapeutic strategies.

Future studies should clarify how defects within individual barrier layers interact with host genetics, environmental exposures, the gut microbiome, and systemic immune responses to influence disease initiation, progression, and treatment response. In particular, well-designed longitudinal human studies, together with adequately powered randomized clinical trials of microbiota-targeted therapies, are needed to establish causality and define the clinical utility of these interventions. Ultimately, a better understanding of these complex interactions may support the development of precision medicine approaches that tailor therapeutic interventions to the specific mechanisms driving inflammation in individual patients with RA.
